# Differences in the choice of beef artificial insemination sires used by dairy producers versus beef producers

**DOI:** 10.3168/jdsc.2023-0426

**Published:** 2023-11-17

**Authors:** D.P. Berry, S.C. Ring, A.J. Twomey

**Affiliations:** ^1^ Teagasc, Animal & Grassland Research and Innovation Centre, Moorepark, Fermoy P61 P302, Co. Cork, Ireland; ^2^ Irish Cattle Breeding Federation, Shinagh, Bandon P72 X050, Co. Cork, Ireland

## Abstract

The growing demand among dairy producers for suitable beef sires to mate to their females creates the possibility of separate breeding programs to generate beef sires for the dairy sector versus those for the beef sector. Informing such a decision is the extent of the genetic differences among beef sires used by dairy producers relative to those used by beef producers. The objective therefore of the present study was to use a large national database of artificial insemination (AI) records in dairy and beef cow herds to establish the difference in mean genetic merit of beef AI sires used by dairy producers versus those used by cow-calf beef producers. The traits explored were gestation length, calving difficulty, and perinatal mortality as well as the 3 carcass traits of carcass weight, conformation, and fat score. Carcass conformation and fat score are mechanically assessed on a scale of 1 (poor conformation and low fat cover) to 15 (excellent conformation and high fat cover). Sire genetic merit differences for feed intake and docility were also examined. Estimates of genetic merit for all 8 traits on individual AI sires available at the time of service were used. A total of 1,230,622 AI records comprised 909,719 services from dairy herds and 320,903 services from beef herds were used. Of the 1,802 beef AI sires represented in the entire dataset, over half were used by both dairy and beef herds representing ≥98% of the services in each production system. However, the usage rate of individual AI sires differed between dairy and beef herds with the Spearman rank correlation between the quantity of inseminations per sire in dairy and beef herds being just 0.38. This correlation means that beef AI sires used heavily in the beef herd were not always those heavily used in dairy herds. A clear difference in the mean genetic merit of beef AI sires selected by dairy producers relative to those selected by beef cow-calf producers was obvious with the extent of the difference being a function of whether the female served was a nulliparous heifer or a cow. Much of the differences in genetic merit of chosen beef AI sires between dairy and beef producers was actually attributable to differences in breed choice, albeit some within-breed selection was also evident. Irrespective, dairy producers, on average, chose shorter gestation length sires whose progeny were genetically less predisposed to require intervention during the birthing process; these sires had genetic merit estimates expected to result in lighter and less conformed progeny carcasses relative to the beef AI sires used by beef producers. Results point to large differences in genetic merit of the beef AI sires chosen by dairy versus beef producers, much of which actually reflected differences in breed choice among dairy and beef producers.

## Highlights

• More than half of the beef AI sires used were common to both dairy and beef herds.
• Differences in genetic merit of chosen beef AI sires were mainly due to breed choice.
• Dairy producers chose beef AI sires with shorter gestation length and easier calving.
• Beef AI sires used by dairy producers had genetically inferior progeny carcasses.

## Graphical Abstract

**Figure:**
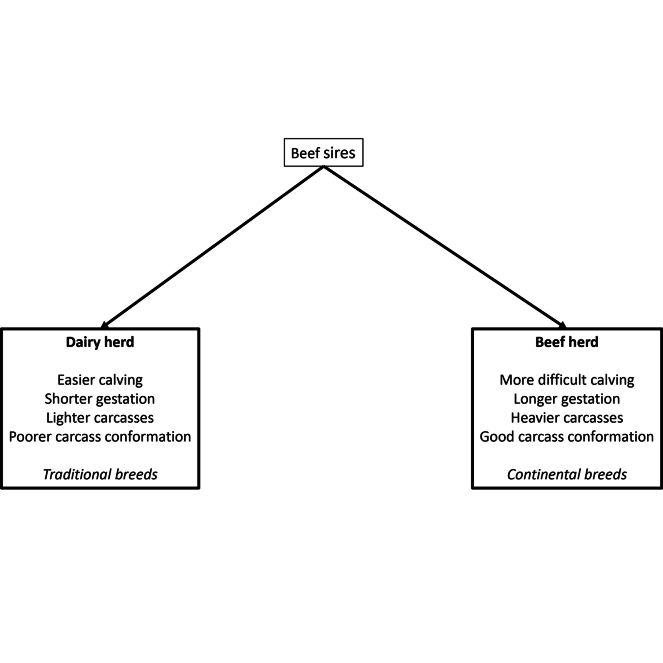
**Summary** Beef sires used by dairy producers differ from those used by beef producers, but information on the extent of this difference per trait is lacking. Using a large national database of artificial insemination (AI) records from dairy and beef cattle, this study demonstrated that relative to beef producers, dairy producers use, on average, beef AI sires that are expected to produce shorter-gestation progeny requiring less intervention at birth. The genetic merit of these beef AI sires, however, resulted in progeny with lighter and less conformed carcasses relative to the progeny of beef AI sires used by beef producers.

Choice of sire is a recurring and critical decision on both dairy and beef farms; the preference is often affected by the (estimated genetic) attributes of the sires, including breed, as well as other factors such as semen supplier, availability, and cost. Understanding such choices can provide useful insights into the mindset of producers aiding the development of extension campaigns and informing tailored decision support; such details can also inform the need for bespoke breeding programs. For example, a survey of Irish dairy and beef producers highlighted that the requirement for intervention at calving was more a concern for dairy than beef producers (Martin-Collado et al., 2017). This implies that attention to sire genetic predisposition to calving difficulty may be more important for dairy than beef producers. Using a large national database on the genetic merit of AI sires used in Irish dairy herds, Berry et al. (2020) compared the mean genetic merit of both dairy and beef sires used by dairy producers for a series of traits. The beef AI sires used in the dairy herds had progeny that, on average, were genetically more at risk of requiring intervention at birth compared with the progeny of the dairy sires used (Berry et al., 2020). The genetic merit of the beef AI sires though were expected to deliver heavier and more conformed progeny carcasses relative to the progeny of the dairy sires used in those herds (Berry et al., 2020). What was not investigated in the study of Berry et al. (2020), however, was how the genetic merit of the beef sires used by the dairy producers compared with the genetic merit of beef AI sires used by beef producers. In fact, there is a paucity of studies globally that have compared the features of (AI) beef sires used by dairy producers versus those used by beef producers as well as the frequency with which the same beef sires are used in both dairy and beef herds. One reason for such a dearth of information is that many countries do not provide across-breed genetic evaluations of beef sires to enable a direct comparison of sires across breeds but also the data from dairy and beef herds may not always be readily available in a single repository or common dataset. Ireland operates an across-breed cattle genetic evaluation for a series of traits, and all cattle data (from dairy and beef producers) are stored in a central national repository. The objective, therefore, of the present study was to fill the cited gap in knowledge by undertaking a cross-sectional analysis of the Irish national database of dairy and beef herds across a 7-yr period. This research question has become more topical in recent years given the growth in the market demand among dairy producers for suitable beef sires (Berry, 2021; Basiel and Felix, 2022). This poses questions for breeding programs as to whether specialized lines or breeds are necessary for dairy producers and beef producers separately. Knowing the extent of the difference in the type of beef AI sire used by both sectors can therefore help populate models attempting to optimize beef breeding schemes.

Predicted transmitting abilities from the last national genetic evaluation of each of the years 2013 to 2019 inclusive for direct gestation length (days), direct calving difficulty (percent requiring considerable assistance or more), direct perinatal mortality (percent) as well as carcass weight (kg), conformation (1 [poor] to 15 [excellent]), fat score (1 [light fat cover] to 15 [heavy fat cover]), docility (1 [flighty] to 5 [docile]), and feed intake (kg DM/d) were available for beef AI sires. Carcass conformation score reflects the shape and development of the carcass, with emphasis on the round, back and shoulders. Carcass fat reflects the level of fat covering the carcass as well as within the thoracic cavity. Neither the genetic evaluation models nor the genetic evaluation base population changed over this time period. All genetic evaluations for these traits are across-breed including all dairy and beef breeds; all generated PTA are expressed relative to a common base population and thus are comparable across breeds. The PTA are expressed in the units of measurement. The beef breeds considered in the present study were Angus, Aubrac, Belgian Blue, Charolais, Hereford, Limousin, Piedmontese, Parthenaise, Salers, Shorthorn, and Simmental.

Artificial insemination records from dairy and beef herds between the years 2014 and 2020 inclusive were available; only AI services of beef sires were retained. To be considered in the study, all herds had to have at least 10 breeding females and at least 10 recorded AI records. Some do-it-yourself AI herds only record the last insemination per cow and these herds were removed (Berry et al., 2020). Only parities up to parity 15 were included and parity was recoded as 0 (i.e., heifers), 1, 2, 3, 4, or 5+. A total of 1,230,622 AI records comprising 909,719 service records from dairy herds and 320,903 services from beef herds were used. These services were from 481,671 females in 46,198 dairy herd-years and 123,781 females in 25,543 beef herd-years. The service sire PTA for each trait from the national genetic evaluation in the months immediately before when the service occurred was available; this represents the PTA available to the producer close in time to when the choice was made of which AI sire(s) to use.

Linear fixed effects models were used to quantify the association between the dependent variable and whether the herd was dairy or beef. The dependent variable considered was the PTA of the AI sire for each trait separately. The categorical (independent) variable of interest was whether the insemination was undertaken in a dairy or a beef herd. Whether the association between herd type and PTA of the beef AI sire differed by parity was also investigated. Preliminary analyses revealed little difference, within herd type, in the mean PTA of the beef AI sires mated to cows (i.e., parity ≥1) of different parities; therefore, parity in the analysis was defined as simply nulliparous heifers versus cows. In a supplementary analysis, breed of the sire was also included as a categorical variable in the model to investigate if within-breed differences in the AI sire(s) chosen existed between herd types.

In a further analysis exploring the association between herd type and sire breed choice, sire breed was dichotomized into either traditional (i.e., Angus, Hereford, and Shorthorn) or others. This binary variable was then considered to be the dependent variable and the association between herd type and the logit of sire breed category was determined using logistic regression assuming a binomial distribution of the errors.

The median herd size of the dairy and beef herds used in the dataset was 74 and 22, respectively. However, these do not represent national statistics because herds included in the present study were those remaining after all data edits, in particular the requirement to have used beef AI. Of the beef-sired calves born in dairy herds in the year 2020 (unedited dataset), 57% had a recorded sire and, of these, 36% were sired by AI. Of the beef-sired calves born in beef herds in the year 2020 (unedited dataset), 80% had a recorded sire and, of these, 22% were sired by an AI sire. At least anecdotally, it could be assumed that where sires are unrecorded, they are generally not served by an AI sire. The rationale for restricting the analysis to just AI sires was that choice of natural mating sires can be influenced by more external factors such as the bulls available at the particular sale, the asking price of those bulls, and the competition with other potential purchasers, which could affect the ability to acquire the desired bull.

A total of 1,802 beef AI sires were represented in the entire dataset, of which 923 were common to both dairy and beef herds. The services to these 923 sires represented the overwhelming majority of all services accounting for 895,948 of the AI services in dairy herds (98% of all services in the edited dataset) and 314,250 of the AI services in beef herds (98% of all services in the edited dataset). A total of 486 beef bulls were exclusively used in dairy herds of which 18% were Angus, 20% were Simmental, and 16% were Hereford. A total of 393 beef bulls were exclusively used in beef herds (for the period under study) of which 27% were Charolais, 22% were Limousin, 16% were Simmental, and 5% were Angus. Despite this obvious use of the same beef AI sires in both dairy and beef herds, the Spearman correlation between the number of serves per sire in dairy and beef herds was just 0.38, signifying that that relative usage rate differed by herd type. This indicates that different criteria are used when selecting beef AI sires for use in dairy versus beef herds. This is not unexpected because beef producers are recommended to choose sires based on a terminal index designed specifically for beef females (Connolly et al., 2016), whereas dairy producers are recommended to choose sires based on a beef-on-dairy breeding index specifically designed to identify suitable sires for mating to dairy females (Berry et al., 2019). The correlation between the terminal index and dairy-beef index values of 309 high-reliability AI Angus bulls in Ireland was 0.68. This value is, nonetheless, stronger than the minimum (genetic) correlation of 0.60 suggested by Mulder et al. (2006), suggesting that different breeding programs for beef and dairy females are not justified.

The mean genetic merit of the beef sires used in dairy herds relative to those used in beef herds (i.e., the reference category) is in Table 1. Clearly, dairy producers actively preferred beef AI sires with expected shorter gestations and where progeny are expected to require less assistance at birth; these differences between both herd types represent >2 standard deviation units in the variability of PTA of all beef AI sires used in the entire population for gestation length and almost 1 standard deviation for calving difficulty. The significance of the interaction term in the model (*P* < 0.001) between herd type and parity pointed to the fact that the difference in mean PTA of beef sires for gestation length and calving difficulty differed by whether the female mated was a heifer or a cow (Table 2). The mean difference in gestation length PTA of the beef sires when mated to heifers in beef or dairy herds was 0.76 d greater than the mean difference in gestation length PTA of the beef AI sires when mated to cows in beef or dairy herds. In contrast, the mean difference between herd types in the calving difficulty PTA of the beef sires used was greater in cows than in heifers. Nonetheless, this interaction effect was actually biologically small for both gestation length and calving difficulty. Moreover, the difference in calving difficulty PTA of the beef sires mated to heifers versus cows was greater in beef herds than in dairy herds; irrespective of herd type, beef sires genetically less prone to their progeny requiring assistance during the birthing process were mated to nulliparous heifers as a strategy to mitigate the greater risk of calving dystocia in heifers (Dematawewa and Berger, 1997). Beef sires mated to dairy females had a lower genetic predisposition to perinatal mortality that those mated to beef females (Table 1) with the difference being greater in cows than in heifers (significance of the interaction term was *P* < 0.001; Table 2).

**Table 1:** Standard deviation in PTA of the 1,802 beef AI sires used (SD) for each trait along with the mean difference (SE in parentheses) between the PTA of the beef AI sires used in dairy herds versus those used in beef herds without (Without) or with (Adjusted) adjustment for differences in sire breed used

Trait	SD	Without	Adjusted
Gestation (d)	1.16	−2.72 (0.003)	−0.44 (0.003)
Calving difficulty (%)	2.50	−2.35 (0.004)	−1.09 (0.003)
Mortality (%)	0.53	−0.30 (0.001)	−0.21 (0.001)
Carcass weight (kg)	7.98	−16.96 (0.018)	−3.22 (0.015)
Carcass conformation^1^ (units)	0.37	−0.97 (0.001)	−0.18 (0.001)
Carcass fat^1^ (units)	0.24	0.51 (0.001)	0.02 (0.001)
Docility^2^ (units)	0.08	0.03 (0.0002)	−0.02 (0.0002)
Feed intake (kg/d)	0.19	0.19 (0.0005)	−0.03 (0.0004)

1 Scored on a scale of 1 to 15 (poor to good conformation and lean to fat carcass score).

2 Scored on a scale of 1 (flighty) to 5 (docile) score.

**Table 2:** Relative mean (SE in parentheses) PTA per trait of the beef AI sires mated to dairy heifers, dairy cows, and beef cows when compared with those mated to beef heifers without (Without) or with (Adjusted) adjustment for differences in sire breed used

Trait	Without			Adjusted		
	Dairy heifers	Dairy cows	Beef cows	Dairy heifers	Dairy cows	Beef cows
Gestation (d)	−3.49 (0.01)	−1.53 (0.01)	1.19 (0.01)	−0.46 (0.01)	−0.06 (0.01)	0.42 (0.01)
Calving difficulty (%)	−1.93 (0.01)	0.34 (0.01)	2.83 (0.01)	−0.42 (0.01)	−0.06 (0.01)	1.18 (0.01)
Mortality (%)	−0.19 (0.003)	−0.01 (0.003)	0.30 (0.003)	−0.11 (0.003)	−0.004 (0.003)	0.24 (0.003)
Carcass weight (kg)	−15.11 (0.05)	−7.17 (0.04)	10.39 (0.04)	−4.48 (0.03)	−0.36 (0.03)	3.13 (0.03)
Carcass conformation^1^ (units)	−0.89 (0.003)	−0.55 (0.003)	0.44 (0.003)	−0.06 (0.002)	−0.07 (0.001)	0.12 (0.001)
Carcass fat^1^ (units)	0.47 (0.002)	0.24 (0.002)	−0.28 (0.002)	0.004 (0.001)	−0.01 (0.001)	−0.03 (0.001)
Docility^2^ (units)	0.01 (0.001)	0.08 (0.0004)	0.05 (0.0004)	−0.02 (0.0005)	−0.01 (0.0004)	0.01 (0.0004)
Feed intake (kg/d)	0.27 (0.002)	0.19 (0.001)	0.02 (0.001)	−0.13 (0.001)	−0.01 (0.001)	0.02 (0.001)

1 Scored on a scale of 1 to 15 (poor to good conformation and lean to fat carcass score).

2 Scored on a scale of 1 (flighty) to 5 (docile) score.

The fact that dairy producers, on average, favor beef sires with expected shorter gestation and easier calving is not surprising (Martin-Collado et al., 2017) with the latter likely also translating to less perinatal morality given the known genetic association between calving difficulty and perinatal mortality (Hansen et al., 2004). Calving date is an economically important trait in seasonal-calving herds (Shalloo et al., 2014) such as those in Irish dairy (Berry et al., 2013) and beef (Berry and Evans, 2014) production systems. Because dairy cows in seasonal-calving systems are often dried off based on a calendar date, a delay in calving date translates to a shorter lactation, which has obvious ramifications for milk receipts. Therefore, gestation length has a strong economic value (Berry et al., 2019), especially in seasonal dairy herds which predominate in Ireland. The milk yield of dairy cows who experience assistance or dystocia at calving is well known to be reduced (Berry et al., 2019; Dematawewa and Berger, 1997) thereby also affecting profit (Berry et al., 2019). Dystocia also affects subsequent cow fertility (Dematawewa and Berger, 1997; Berry et al., 2019) and thus calving date in the following calving season. Therefore, it stands to reason that both gestation length and calving dystocia are a priority for dairy producers when selecting sires. This is especially true for beef sires in Ireland who tend to be used in the latter period of the breeding season (Berry et al., 2020) when subsequent gestation length and calving difficulty are more important in the pursuit of a compact calving season. Gestation length and calving difficulty are also undoubtedly important in cow-calf beef herds, but their priority during sire selection relative to other traits (e.g., carcass merit) may differ.

Because of the difference in herd size between beef and dairy herds used in the analysis, a supplementary analysis was undertaken limiting the dairy herd population to just the 9,380 dairy herd-years with <40 cows calving (median herd size was 29). There was no impact on the study conclusions for any of the traits investigated. For example, the mean difference in beef sire PTA between dairy and beef herds for gestation length changed from 2.72 d in the full dataset to 2.57 d in the restricted dataset, whereas the corresponding values for direct calving difficulty PTA were 2.35% and 2.16%, respectively.

When, however, the PTA of the beef AI sires used by dairy and beef producers was expressed within breed (i.e., when sire breed was included as a fixed effect in the model), the differential in PTA of the chosen beef sires between herd type reduced considerably (Table 1). For example, the mean gestation length PTA of beef AI sires used by dairy farmers reduced from being 2.72 d shorter than beef herds when considered across breeds to just 0.44 d (shorter gestation was still preferred by dairy producers); this implies that much of the observed difference between herd types in the mean PTA of the beef AI sires chosen was actually due to between-breed selection, albeit some within-breed selection was also practiced. In fact, when the beef sire breeds were categorized into traditional (i.e., Angus, Hereford, Shorthorn) versus continental (i.e., other breeds), the odds of a traditional breed sire being used by dairy producers was 22.0 times (95% CI: 21.7 to 22.2) more than that of being used by beef cow-calf producers; this odds increased to 29.6 (95% CI: 29.2 to 30.1) when parity was included in the model. A greater use of traditional breed beef sires in dairy herds relative to continental sires has already been reported in Irish dairy herds (Berry and Ring, 2020a), albeit this trend is not consistent across all countries (Davis et al., 2019). The traditional breeds are known to, on average, have shorter gestation and easier calving that the continental breeds (Berry and Ring, 2020b), although large intrabreed variability is known to exist. The mean (SD) gestation length PTA of the traditional breed AI sires used in the present study was −1.01 d (1.88 d), whereas that of the continental AI sires was 2.04 d (1.63 d). The corresponding values for calving difficulty PTA (more positive values translate to more expected difficulty at calving) of traditional breed sires and continental breed AI sires was 2.65% (1.38%) and 6.42% (2.69%), respectively. This difference in calving difficulty PTA equates to an expectation of almost 4 fewer dystocia events per 100 cows in the progeny of traditional breed sires relative to continental breed sires.

Although an interaction between cow parity and herd type existed (*P* < 0.001), the difference in mean carcass PTA of the beef AI sires mated to dairy versus those mated to beef heifers was only marginally smaller than the respective difference between the beef sires mated to dairy and beef cows (Table 2). Hence, only the main differences in beef sire PTA between dairy and beef herds are discussed further, ignoring the interaction with parity, which is nonetheless presented in Table 2. The mean PTA of the beef sires used in dairy herds for carcass weight was 16.96 kg lighter than those used in beef herds, whereas that for conformation PTA was almost 1 unit less (i.e., worse) on the 15-point scale (Table 1); based on the standard deviation in the respective PTA among the 1,802 beef sires used the present study, these differences in carcass weight and conformation represent >2 standard deviation units. The mean PTA for carcass fat was greater (i.e., more fat) for the beef sires used in dairy herds relative to those used in beef herds (Table 1). Whether these observations hold in other populations is not known, although the observed trends may also persist for other carcass-related traits. Moderate to strong phenotypic (Judge et al., 2019a) and genetic (Judge et al., 2019b) correlations have been reported between carcass EUROP conformation score and primal cut weights in cattle. Similarly, Kelly et al. (2019) reported a positive phenotypic (0.43) and genetic (0.60) correlation between carcass EUROP conformation score and ultrasound muscle depth in live cattle. Also investigated in the present study was PTA for docility and feed intake because they form part of the national beef breeding indexes for both dairy and beef in Ireland (Berry et al., 2019). Small differences in feed intake and docility PTA of the beef AI sires used in dairy and beef herds were evident (Table 1), although these differences are unlikely to be due to direct selection by producers but instead an indirect result of conscious selection decisions on calving and carcass-related traits.

Like for the calving performance traits, the observed differences between herd types in sire carcass PTA was largely due to differences in the breeds used with the mean PTA of the sires used in dairy and beef herds converging once sire breed was included in the statistical model. Nonetheless, even within breed, dairy producers used beef AI sires that were genetically expected to have lighter and less conformed carcasses than their beef producer counterparts. It is unlikely that dairy farmers actively select beef sires with poor carcass credentials, so this observation is more likely a function of the dairy producers actively selecting easier calving sires who, because of the genetic correlations between direct calving difficulty and carcass traits are, on average, lighter and less well conformed (Eriksson et al., 2004). Most dairy farmers tend to sell surplus calves soon post-birth (Berry and Ring, 2021) where within-breed differences in genetic merit of the sire for carcass credentials are not often obvious; the value of the calf therefore is often affected by its breed composition (Mc Hugh et al., 2010) as well as other nongenetic features such as age, visual health status, and the prevailing market. Even at that, beef output represents only ca. 5% of the gross revenue or income in many dairy herds (Berry, 2021). One option to incentivize dairy farmers to be more cognizant of the expected beef merit of the calf when choosing beef sires is to attribute an expected profit value to each calf at the point of sale, as suggested by Dunne et al. (2021)—dairy × beef progeny from sires with superior carcass genetic merit credentials should excel in such a system commanding a higher price at sale.

The motivation for the present study was to quantify the actual differences between dairy and beef producers in genetic merit of the beef AI bulls selected for a series of traits. Of particular interest were the traits associated with calving performance and carcass merit. In conclusion, results from the present study demonstrate a large difference in the mean genetic merit of beef AI sires selected by dairy producers relative to those selected by beef producers with the extent of the difference being a function of the parity of the cow. In many cases, the differences between herd types in the genetic merit of the beef AI sires used was in excess of 2 standard deviations of the entire beef sire population used. Much of this observed difference in the genetic merit of chosen sires by dairy and beef producers was actually due to selection between breeds albeit some within-breed selection was also evident. Irrespective, dairy producers favored shorter gestation beef sires that were genetically less predisposed to calving assistance; this manifests itself through genetic correlations as less mortality and lighter and less conformed carcasses. The challenge for beef seedstock breeders is to locate sires suitable for mating to dairy females (Berry et al., 2019) which, if identified using a selection index, could improve carcass credentials without any concomitant deterioration in gestation length or calving difficulty genetic merit. Results from the present study are, nonetheless, based on only Irish data where a strong integration between the dairy and beef sectors exists. Whether identified trends exist in other populations certainly warrants investigation so as to help inform the development of beef breeding programs.
